# The physiological mechanism and effect of resistance exercise on cognitive function in the elderly people

**DOI:** 10.3389/fpubh.2022.1013734

**Published:** 2022-11-22

**Authors:** Aijie Cheng, Zhiwei Zhao, Hengxu Liu, Jinxin Yang, Jiong Luo

**Affiliations:** The Institute of Sports Rehabilitation, Southwest University, Chongqing, China

**Keywords:** Alzheimer's disease, elderly, cognitive health, physiological function, resistance exercise

## Abstract

**Background:**

As brain function declines and cognitive ability declines, the benefits of resistance exercise to the brain of older people are gradually gaining attention.

**Objective:**

The purpose of this review is to explore the mechanism and relationship between physiological factors such as vascular and neuronal degeneration and cognitive decline, and to categorize the differences in the effects of an acute and chronic resistance exercise intervention on cognitive function in healthy elderly people and the possible regulators of cognitive effects.

**Methods:**

Using PubMed, Elsevier, Web of Science, X-MOL, CNKI, and Taiwan academic literature database, the research papers published in relevant journals at home and abroad until April 2022 were searched with Chinese and English keywords such as Resistance exercise, the elderly, hippocampus, memory performance, neurons, cognitive function. Pedro scale was used to check the quality of various documents, and the relevant research documents were obtained with the resistance exercise elements as the main axis for comprehensive analysis.

**Results and conclusion:**

(1) Resistance exercise can have a beneficial effect on the brain function of the elderly through blood flow changes, stimulate nerve conduction substances and endocrine metabolism, promote cerebrovascular regeneration and gray matter volume of the brain, and prevent or delay the cognitive function degradation such as memory and attention of the elderly; (2) Acute resistance can temporarily stimulate hormone secretion *in vivo* and significantly improve the effect of short-term memory test, but it has little effect on the cognitive performance of the elderly; (3) Moderate-high intensity resistance exercise (50–80%1RM, 1–3 times/week, 2–3 groups/time) lasting for at least 6 months is more prominent for the improvement of cognitive function of the elderly, while the parameters such as resistance exercise intensity, exercise amount, duration, evaluation test time and differences of subjects may have different degrees of influence on cognitive benefits.

## Introduction

According to the official demographic forecast, China's elderly population will reach 480 million in 2050 (1). With the increase of age, physical abilities (such as muscle strength) and cognitive functions (such as attention, memory, and central executive control capability) will gradually decline (2). The structure and function of the organic system (i.e., brain) may be negatively affected, leading to a decline in individual abilities (e.g., cognitive ability), and ultimately leading to Alzheimer's disease (AD). Cellular and molecular biological studies have shown that brain-derived neurotrophic factor (BDNF) and cortisol (COR) have an impact on the neurogenesis of the brain of all mammalian species (including humans), which directly change the basic structure and morphology of the brain (3). In addition, the study found that cerebrovascular dysfunction, neuronal plasticity, neurogenesis, and stress hormone levels are all related to the etiology of AD or cognitive and emotional disorders (4).

Notably, resistance exercise (RE) can stimulate a variety of neurochemicals, such as lactate, cortisol (COR), brain-derived neurotrophic factor (BDNF), serum insulin-like growth factor-1(IGF-1), vascular endothelial growth factor (VEGF), acetylcholine, dopamine, norepinephrine, and serotonin (5). These specific neurochemicals interact with each other through other regulatory factors, triggering complex neurobiological processes (6–8), and making elastic changes in cognitive control through environmental changes. They can also inject more attention resources to adjust behavioral performance after reaction errors or conflicts (9, 10). At the same time, it can also fight against different types of body and brain-related health problems, such as angiogenesis, which can enhance blood perfusion, learning-related hippocampus, and chronic memory (11).

Given that there is currently no cure for cognitive decline, and that the health and economic burden of a general decline in people's cognition has seriously affected social life. Therefore, establishing prevention methods is critical to delaying the occurrence of cognitive decline. However, structured resistance exercise has been shown to improve memory, attention, spatial awareness, reaction time, planning, and information processing capabilities in middle-aged and older adults (12). To date, there is evidence to support the close relationship between neurodegenerative diseases and progressive cognitive decline during aging, but its exact physiological mechanism is still unclear. And there is limited information about the different benefits of acute resistance or chronic resistance intervention on the cognitive health of the elderly. Therefore, the primary end-point of the review is to explore the mechanisms and relationships between physiological factors such as vascular and neuronal degeneration and cognitive decline. Furthermore, we also discuss the difference in the effectiveness of acute and long-term resistance exercise intervention on the cognitive function of healthy elderly people, to provide an important reference for future academic research or training practice.

## Methods

### Search strategy

We conducted a comprehensive literature search in databases including PubMed, Elsevier, Web of Science, X-MOL, CNKI, and Taiwan academic literature for reports published from inception through the date of search (initial search conducted on November 3, 2021, with an updated search conducted on April 7, 2022). Searches were specific to literature published in English and Chinese but were not limited to any age or publication date ranges.

### Literature selection

**Figure 1:**
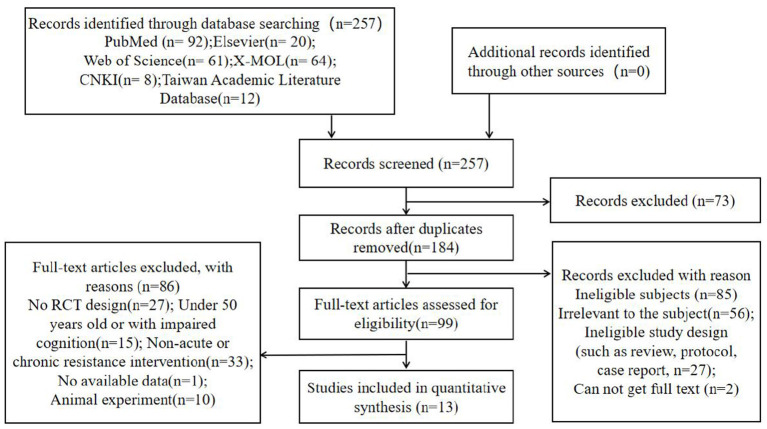
Flow chart with information about the search, screening, and selection processes.

We identified 3,502 studies from the different searched databases based on “elderly” and “cognitive function,” and from these 3,502 studies identified 1827 studies based on the keyword “resistance exercise” or “resistance training.” Then, a total of 257 studies were further identified by the relevant terms, including specifically “acute resistance” or “chronic resistance” and terms related to cognition in older adults (atherosclerosis, hippocampus, memory performance, neurons) and “Alzheimer's disease.” Among the 257 pieces of related literature established, 184 records were retained by removing duplicate records. Two reviewers (AC, HL) carefully read the abstracts and contents of the remaining articles, and then excluded the articles irrelevant to the subject (*n* = 56), ineligible study design (such as review, protocol, case report, *n* = 27), and cannot get full text (*n* = 2). Disagreements were resolved through consensus or with the help of a third reviewer (JL). Pedro Scale was used to check and judge the literature quality, and finally, 99 pieces of literature were obtained. The details of our study selection process are reported in Figure 1.

### Data extraction and analysis

One reviewer (AC) used a specially designed form to extract data from the included research, and it was verified by another reviewer (HX). The extracted data contained basic information about the publication (article title, author, year of publication, journal name, etc.), design (randomization, allocation concealment, etc.), characteristics of subjects (number, age, sex, etc.), intervention measures (intervention type, frequency, intensity and duration of each group) and outcomes (measurement tools, drop-out, etc.) were extracted from each included study. Among them, 13 items (5 acute resistance and 8 chronic resistance) were included in the quantitative comprehensive analysis, and the remaining 86 articles were excluded for the following reasons: (1) No RCT design (*n* = 27); (2) Under 50 years old or with impaired cognition (*n* = 15); (3) Non-acute or chronic resistance intervention (*n* = 33); (4) No available data (*n* = 1); (5) Animal experiment (*n* = 10).

Cochrane Review Manager (RevMan 5.4) was used to perform the data analysis. Mean difference (MD) or standardized MD (SMD) with 95% confidence intervals (95%CIs) were employed to measure trial outcomes of continuous data. If the data are available and the heterogeneity test does not find significant heterogeneity among the included studies, the fixed effect model is used to estimate the merger effect; Otherwise, the random effect model is used.

### Risk of bias assessment

**Figure 2:**
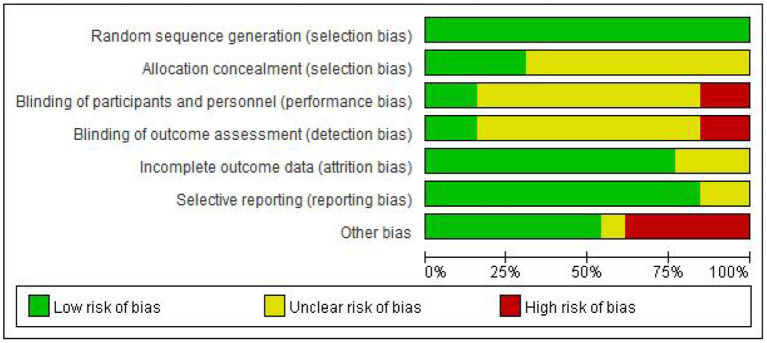
Risk of randomized control studies bias graph.

**Figure 3:**
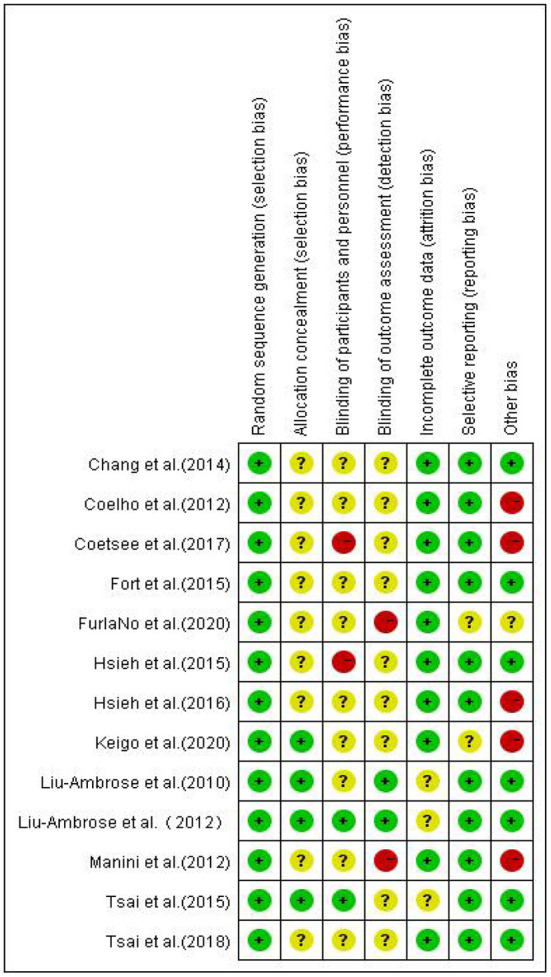
Risk of randomized control studies bias summary.

Two evaluators independently evaluated the research quality by using Cochrane Collaboration's Risk of Bias Tool. The evaluation criteria of this tool are divided into seven items, and the bias risk rating of each item is “low,” “high” or “unclear” to judge and divide the research quality (13). Any differences in bias risk assessment are resolved through discussions between evaluators or with the third author when necessary. The evaluation results are reported in Figures 2, 3.

It was found that in the blind field of Random sequence generation, Allocation concealment, incomplete outcome data, and Selective reporting, most studies are rated as low bias risk or unclear bias risk. The reviewed studies are considered to have ambiguous bias risks in these areas because they are not described in sufficient detail (such as allocation concealment). However, there are high-performance bias risks in the other three blind areas, which is inevitable.

## Mechanism of aging and neurodegenerative diseases on cognitive health

### Cerebral-vascular dysfunction

#### Atherosclerosis, cerebral blood flow

Atherosclerosis (AS) is a vascular disease with the hardening of blood vessels, internal thickening, and inhibition of vascular tension (14). It is characterized by the formation of atherosclerotic plaque or fibrous plaque in the intima, and it is one of the most common and dangerous diseases (15). After developing AS the atherosclerotic carotid and cerebral arteries attenuate cerebral blood flow (CBF) (16) over time. The decrease in cerebral blood flow is related to insufficient cerebral perfusion, reduced nutrient transport, low metabolism, and cognitive ability, and it decreases by 1% in healthy adults every year (17). The Rotterdam study (18) showed that a patient with severe atherosclerosis has three times the probability of developing AD compared with normal people. In the process of AS formation, under the condition of a high-fat diet (high cholesterol and saturated fat), extracellular lipids and white blood cells accumulate in the intima, and endothelial cells produce relatively reduced shear stress at the proximal arterial blood flow shunt (19), which leads to abnormal hemodynamics and increased blood viscosity. It is found that atherosclerosis is positively correlated with blood viscosity (20). Compared with people with normal blood viscosity, older men with higher blood viscosity have poorer cognitive performance (21). Importantly, blood viscosity increases with age (22), and is affected by erythrocyte aggregation, leukocyte count, plasma fibrinogen, immunoglobulin, and erythrocyte rigidity (23). In the presence of cognitive impairment, these age-related rheological changes will be more obvious (24). For example, fibrinogen concentration increases with age, and it is related to the cognitive decline of individuals with mild cognitive impairment (RP = 0.17, *p* < 0.05) (25). Lucas et al. (26) pointed out that the increase in blood viscosity and the decrease of CBF related to age reflect brain atrophy because less active nerve mass requires less oxygen and nutrition (i.e., low metabolism), so it is speculated that low metabolism may be one of the reasons for the decrease of CBF. In addition, the change in blood pressure is speculated to be one of the key factors to reduce CBF. At present, the relationship between hypotension and cognition is unclear, but hypertension can indirectly reduce CBF by destroying the brain's self-regulation, promoting harmful vascular remodeling, oxidative stress, and inflammatory response (27), and paving the way for white matter degeneration.

**Figure 4:**
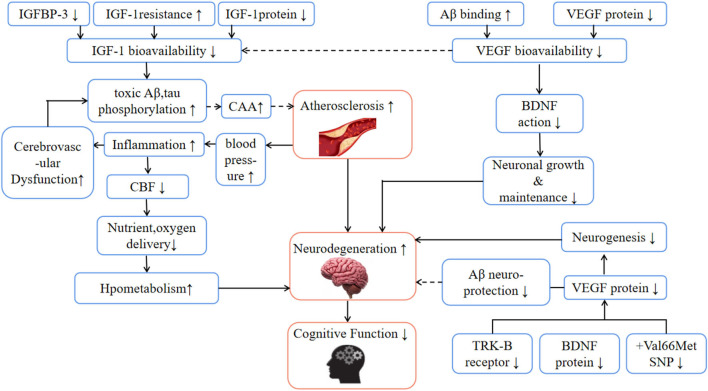
The effects of aging and neurodegenerative diseases on physiological mechanisms related to cognitive health. Aβ, amyloid β; BDNF, brain-derived nerve factor; CAA, cerebral amyloid angiopathy; CBF, cerebral blood flow; IGF-1, insulin-like growth factor 1; IGFBP-3, insulin-like growth factor binding protein 3; TRK-B, tropomyosin-related kinase b; SNP, single nucleotide polymorphism; VEGF, vascular endothelial growth factor; Hypothetical pathways are marked “—”.

In addition, inflammatory factors (such as apolipoprotein B- lipoprotein (apoB-LPs), interleukin IL-1, IL-6, tumor necrosis factor-α, etc.), embryonic phenotype smooth muscle cells (SMC), and arterial extracellular matrix (ECM) in AS lesions exacerbate the plaque inflammation and calcification (28). Calcification not only causes the dysfunction of the blood-brain barrier (BBB) but also accelerates the deposition of amyloid-β(Aβ) in and around cerebral arteries. This condition is called cerebral amyloid angiopathy (CAA), and its severity is related to the accelerated decline of perception speed, situational memory, and semantic memory of the elderly (29). Generally, Aβ is a landmark feature observed in the brains of AD patients and is considered to be the main cause of neuron degeneration (30). In the pathogenesis of AD, Aβ is strongly supported by the rare gene mutations of transmembrane protein amyloid-β, presenilin 1, and 2, which directly lead to the overproduction of Aβ (31). Due to the unbalanced production and discharge, toxic Aβ peptide is accumulated, which promotes cerebral atherosclerosis (32), and causes vascular dysfunction and permanent circulation accumulation of Aβ (33) (Figure 4). To remove Aβ from the brain, it is necessary to rely on the special way that interstitial fluid (ISF) and cerebrospinal fluid (CSF) are excluded from the cell through BBB and gelatinous lymph (colloid and lymph) system (34, 35). In this process, gelatinous lymphatic drainage occurs along the basilar membrane (perivascular pathway) of arteries and capillaries (36), and the adjacent smooth muscle carrying water channel (34). It is assumed that the movement of ISF and CSF along the perivascular, and the channel depends on arterial pulsation (34, 37). In this model, the space around the blood vessel is compressed during each pulse period, and the resultant pressure wave can remove solutes such as Aβ (38). On the contrary, if the elasticity-dependent arterial pulsation is damaged in AS and aging blood vessels, it is likely that the excretion will decrease and toxic Aβ will accumulate more (34, 35, 39) with time, which will accelerate cognitive decline.

### Neuroplasticity, neurogenesis, neurotrophic growth factors

#### Brain-derived neurotrophic factor

Neuroplasticity is the primary process of remodeling brain structure and function in response to neuron activity, injury, death, and growth (40). As a process of neuroplasticity, neurogenesis is the process of forming neurons from neural stem cells and neural precursor cells. It occurs in three regions of the adult mammalian brain: the subventricular area of the lateral ventricle (for interneuronogenesis), the basolateral region of the amygdala, and the hippocampal dentate gyrus (41, 42). Hippocampal neurogenesis is the way of memory formation, and it is one of the earliest brain regions affected by severe atrophy in AD (43). It exists in the hippocampus and cerebral cortex with a high concentration of brain-derived neurotrophic factor (BDNF). BDNF is the most widely distributed neurotrophic factor in neurogenesis and synaptic plasticity. Through its high-affinity tropomyosin-associated kinase B(TRK-B) receptor (44), BDNF may have a bidirectional transport function across BBB (45), increasing the number of synapses in the brain and the branches of the axonal in the cortex (46). BDNF and IGF-1 are known as the key factors of learning and memory, which are responsible for the generation, proliferation, survival, and maintenance of neurons (such as neurons in the hippocampus, cortex, and root ganglion cells) (47), making the brain operate more efficiently and improving the learning process and memory ability. In addition, *in vivo*, and *in vitro* evidence also shows that BDNF-mediated neuroprotection can inhibit Aβ toxicity in rats (48).

The basic peripheral level of BDNF in healthy individuals varies from 1.5 to 30.9 ng·mL^−1^ (49). Lommatzsch et al. (50) observed that the decrease in plasma BDNF was negatively correlated with the increase in age (*rs* = −0.20, *p* < 0.05). In AD patients, the serum BDNF level rises in the early stage of the disease (about 21%), which may be a compensatory response to mild neurodegeneration (51, 52). In late AD, BDNF has obvious transcription defects in several cortical areas (13), and the level of BDNF protein in serum is 31% lower than that of early AD patients (51). The brain tissue of AD patients after death showed that BDNF mRNA decreased in the crucial areas of memory formation and learning (hippocampus and cortex) (13). In addition, some gene polymorphisms will also harm BDNF. There is a common single nucleotide polymorphism (SNP) in BDNF. At codon 66, the amino acid valine (Val), which is responsible for correctly coding BDNF, is replaced by methionine (Met), a sulfur-containing amino acid (Val66Met SNP). Met substitution damages the activity-dependent secretion and intracellular distribution of BDNF in hippocampal neurons (53). Compared with Val carriers, Met carriers have worse episodic memory and hippocampal function, and the gray matter volume of the prefrontal lobe and temporal lobe is smaller (54).

#### Insulin growth factor (IGF) and growth hormone (GH)

Aging is a dynamic and gradual process, which will decrease the function of the nerve conduction system, reduce the release of nerve substances (such as IGF-1 and GH) (55), and then make cognitive performance decline, reducing the ability of individuals to adapt to the environment. Growth hormone (GH) is the master of all hormones, and it is also the key element for all brain and body cells to grow into adulthood. It can not only control energy allocation but also counteract the natural aging of cells (56). GH mostly exists in the hippocampus, inferior hypothalamus, putamen, and choroid plexus in the brain. However, aging will significantly reduce GH values in these parts of the brain (57). Vitiello et al. (58) speculated that GH was closely related to cognitive function, and GH supplementation could improve the cognitive decline of elderly or AD patients. Insulin-like growth factor 1 (IGF-1) is a hormone produced by GH stimulation, which is secreted by the anterior pituitary gland. After GH secretion, it can promote the synthesis and secretion of IGF-1 by hepatocytes within a few minutes (59). Its main function is the growth and maintenance of skeletal muscle, especially in the developmental stage of life (60). IGF-1 plays an important role in neurogenesis and angiogenesis of the brain. For example, during the transport of IGF-1, it is easy to interact with the target tissue through BBB, which binds insulin-like growth factor protein 3(IGFBP-3) with its high affinity, to produce neuromodulation effects, including assisting in the production of glial cells, myelin sheath, and neurons (61). In addition, IGF-1 may also play an important role in the pathogenesis and chronic neuroprotection of AD. For example, IGF-1 can regulate lipoprotein albumin and thyroxine carrier protein carried by amyloid, and when IGF-1 is inhibited, it will destroy this amyloid regulatory pathway (13). Importantly, a sufficient level of IGF-1 in blood circulation is a necessary condition for pre-BDNF (an immature precursor) (62), which leads to more mature BDNF production. Therefore, IGF-1 is called the intermediary for improving cognitive function after resistance exercise, and it plays a key role in the central nervous system.

### Angiogenesis and vascular endothelial growth factor

Angiogenesis refers to the stimulated growth of capillaries and requires endothelial cell division, basement membrane degradation, and endothelial cell migration to form new blood vessels from the original ones (63, 64). Angiogenesis is physiologically regulated by hypoxia, muscle contraction, fluid shear force, and metabolic changes (65). With the increase of age, the cerebrovascular formation and CBF decrease, the responsiveness to hypoxia decreases, and the expression of vascular endothelial growth factor decreases (66), which leads to vascular dementia. In addition, similar to AD patients, the decrease of angiogenesis in patients with vascular dementia is related to cerebral vasoconstriction, vascular degeneration, and CAA (67).

Vascular endothelial growth factor (VEGF), also known as vascular permeability factor or angiopoietin, is a powerful stimulator of endothelial cell proliferation, germination, and vascular survival, and has the effects of promoting vascular growth and osmotic regulation (68). In addition, VEGF, as a highly specific mitogen, can act on vascular endothelial cells, promote cell proliferation, inhibit their apoptosis, and induce angiogenesis. In AD patients, the serum concentration of VEGF may decrease by 30% (69), and it tends to directly bind to Aβ plaque, which may reduce the bioavailability and brain perfusion of VEGF (70). In the VEGF family (VEGF A-F and placental growth factor), VEGF-A is strongly related to angiogenesis, neurogenesis, and nerve maintenance. For example, angiogenesis induced by VEGF-A (41) can promote nerve regeneration in local tissues of blood vessels. In addition, it was found that VEGF-A stimulated blood vessels can promote the release of BDNF while reducing toxic Aβ deposition and hyperphosphorylation of tau (70). Importantly, the inhibition of VEGF-A will lead to the interruption of neurogenesis, cognitive function, and capillary growth (66). In skeletal muscle, muscle cells are released after stimulation mediated by muscle contraction, and more extracellular VEGF expression depends on the content of interstitial adenosine. Adenosine infusion can up-regulate VEGF of myocardial cells or endothelial cells by interacting with the angiogenic adenosine A2 receptor (70). It has been found that when muscle contraction and blood flow restriction are implemented at the same time (a method for maximizing local muscle hypoxia), the expression of VEGF in serum can be increased by about 150% (71), and neurodegenerative diseases and cognitive decline in the elderly can be reduced. The reason may be that it plays a neuroprotective role by reducing the damage caused by the pathological cascade in patients with cognitive dysfunction, which is beneficial to the integrity of the vascular structure.

## Effect of a resistance exercise intervention on the cognitive function of healthy elderly people

Resistance exercise can not only delay the muscle reduction and degeneration of the elderly, reduce the risk of falls and fractures, but also improve the cognitive function of the brain (72), such as the attention and working memory of the elderly (temporarily storing the current information, and linking and comparing it with subsequent information or events), and impulse control of the young (consciously suppressing the initiation of dominant reactions and behaviors, or suppressing reactions and behaviors that do not meet the current goals) (73). At present, some studies have pointed out that acute aerobic exercise can significantly improve the cognitive function of MCI elderly people, but the effects of an acute and chronic resistance exercise intervention on cognitively healthy elderly people are still different, which will be discussed in the following categories (Tables 1, 2).

**Table 1:** Effect of acute resistance exercise on the cognitive function of the elderly.

**Researcher**	**Research objects**	**Resistance exercise program**		**Research results**	
		**Training content**	**Training design**	**Physiology**	**Cognition**
Tsai et al. (74)	*N* = 66	Biceps bending, triceps stretching, bench pushing, leg pushing, foreleg stretching, butterfly machine pinching.	Number of groups: 2	BDNF↑^a^	Reaction time ↓^b^
	Age:65.3 ± 7 year				
			Strength: 75%1RM	IGF-1↑^b^	
	Sex:28M, 38F	3:RT, AE, CON	Repetitions: 10	VEGF⇔	
			Rest: 90s	FGF-1Δ	
	Condition:MCI				
			Acute		
Manini et al. (75)	*N* = 20	Knee flexion and extension	Number of groups: 4	IGF-1⇔	NS
	Age:67.4 ± 4.6 year (*N* = 10); 28.0 ± 7.8 year (*N* = 10)	2:RT (L), RT (H)	Strength:20%1RM,80%1RM	GH↑^a^	
	Sex:20M		Repetitions: exercise fatigue		
	Condition: Health		Rest: 120s		
			Acute		
Chang et al. (76)	*N* = 30	Biceps left and right bend, high pull-down, bird, bench press, leg bend, leg push.	Number of groups: 2	NS	Expressive force ↑^a^
			Strength: 70%1RM		
	Age:58.1 ± 3.0 year				
	Sex:15F, 15M	2:RT, CON	Repetitions: 10		
	Condition: Health		Rest: 30s, 60s		
			Acute		
Hsieh et al. (77)	*N* = 40	Lying push, leg extension, high pull-down, leg bending, rowing, leg pressing, shoulder pushing, and biceps bending.	Number of groups: 2	NS	Working memory ↑^b^
	Age:21–30 year (*N* = 20); 65–72 (*N* = 20)		Strength: 70%10RM		Processing speed ↑^b^
	Sex:40M	2:RT (1), RT (2)	Repetitions: 10		
	Condition: Health		Rest: 30s, 90s		
			Acute		
Hsieh et al. (78)	*N* = 35	Lying push, leg extension, high pull-down, leg bending, rowing, leg pressing, shoulder pushing, and biceps bending.	Number of groups: 2	NS	Attention ↑^a^
	Age:23.9 ± 2.3 year (*N* = 18); 66.4 ± 1.2 (*N* = 17)				
			Strength: 70%10RM		Reaction time ↑^a^
	Sex:35M	2:RT (1), RT (2)	Repetitions: 10		
	Condition: Health		Rest: NS		
			Acute		

*N*, the number of subjects; H, high load; L, low load; RT, resistance exercise group; HIIT, High-intensity aerobic interval training group; MCT, moderate-intensity continuous aerobic training group; CON, control group; RM, the maximum number of repetitions; BDNF, Brain-derived neurotrophic factor; IGF-1, insulin growth factor-1; FGF-1, fibroblast growth factor-1; MCI, mild cognitive impairment; RM, the maximum number of repetitions; VEGF, vascular endothelial growth factor; a, between-group differences; b, within-group differences;^*^, significant; NS, not specified.

Intra-group differences; ^*^: Remarkable; NS: Not specified.

**Table 2:** Effect of chronic resistance exercise on the cognitive function of the elderly.

**Researcher**	**Research objects**	**Resistance exercise program**		**Research results**	
		**Training content**	**Training design**	**Physiology**	**Cognition**
Keigo et al. (79)	*N* = 50	Legs, stretching sitting posture, leg bending, squat, bench pushing.	Number of groups: 3	NS	Working memory ↑^a^
	Age:50–77 y		Frequency: 3 times/week		Short memory↑^a^
	Sex: NS	3:RT (L), RT (M), CON	Strength: 40%1RM, 60%1RM		Control ↑^a^
			Repetitions: 14		
			Rest: 2min		
			Duration: 24 weeks		
	Condition: Health				
Coelho et al. (80)	*N* = 48	Knee flexion and extension (progressive dynamic resistance training)	Number of groups: 3	BDNF↑^b^	NS
			Frequency: 3 times/week	GNDF?	
	Age:70.5 ± 4.6 year				
	Sex:48F	2: RT (healthy), RT (pre-weak)	Strength	NGF?	
	Condition: Healthy, pre-weak.		First2weeks:50%1RM		
			2–10 weeks: 75%1RM		
			Repetitions: 8		
			Rest: NS		
			Duration: 10 weeks		
FurlaNo et al. (81)	*N* = 24	Progressive resistance (weight) training, balance, and coordination (stretching)	Group number: NS	Hippocampus activation ↑^a^	Memory ↑^a^
					Cognitive expressiveness ↑^a^
	Age:60–80 year		Frequency: 3 times/week		
	Sex: NS	2:RT, BAT	Intensity: increasing gradually.		
			Repetitions: NS		
	Condition: Health		Rest: NS		
			Duration: 6 months		
Fort et al. (82)	*N* = 49	Leg flexion and extension, sitting rowing	Number of groups: 1–2	BDNF↑^a^	NS
	Age:68.0 ± 5.0 year	3:RT (H), RT (H + L), RT (L)	Frequency: 3 times/week		
	Sex:24M, 25FM		Strength:20%1RM(L),20–40%; 1RM(H + L),80%1RM(H)		
	Condition: Health		Repetitions:10–15(H),8–10(L),6–20(H + L)		
			Rest: 60s		
			Duration: 12 weeks		
Tsai et al. (83)	*N* = 48	Biceps bend, leg push, triceps extension, high pull-down, lift heel, sitting rowing.	Number of groups: 3	homocyst	Executive function ↑^a^
			Frequency: 3 times/week	-eine↓^a^	accuracy↑^a^
	Age:71.4 ± 3.8 year				
	Sex:48M	2:RT, CON	Strength: 75–80%1RM	IGF-1↑^a^	Reaction time ↑^a^
			Repetitions: 10	GH?	
			Rest: 90s		
	Condition: Health		Duration: 12 months		
Liu-Ambrose et al. (84)	*N* = 52	Biceps bending, triceps stretching, sitting boating, high drop-down, leg pushing, squat, lunge walking.	Number of groups: 2	White matter atrophy ↓	Cognitive expressive ↑
			Frequency: 1/week, 2/week		
	Age:69.2 ± 3.0 year				
	Sex:52F	3:BAT, RT (1)RT (2)	Strength:7RM(gradually increasing)		
			Repetitions: 6–8		
			Rest: NS		
			Duration: 52 weeks		
	Condition: Health				
Coetsee et al. (85)	*N* = 67	Upper and Lower body muscles (equipment and free weight)	Number of groups: 3	Hemoglobin ↓	Reaction time ↑
			Frequency: 3 times/week		
	Age:62.7 ± 5.7 year				
	Sex:21M, 46F	4:RT, MCT, HIIT, CON	Intensity:50, 75, 100%10RM (first 8 weeks) 75, 85, 100%10RM (last 8 weeks)		
	Condition: Health				
			Repetitions: 10		
			Rest: NS		
			Duration: 16 weeks		
Liu-Ambrose et al. (86)	*N* = 155	Biceps, triceps extension, sitting rowing latissimus dorsi pull-down, kick trainer, leg curl, lift the heel.	Number of groups: 2	NS	Execute control ↑^a^
			Frequency:1/week, 2/week		Attention↑^a^
	Age:65–75 year				
	Sex: Female	2:RT (1), RT (2)	Strength: 70–80%1RM		Conflict resolution ↑^a^
			Repetitions: 6–8		
			Rest: NS		
			Duration: 12 months		
	Condition: Health				

*N*, the number of subjects; H, high load; L, low load; RT, resistance exercise group; HIIT, High-intensity aerobic interval training group; MCT, moderate-intensity continuous aerobic training group; CON, control group; RM, the maximum number of repetitions; BDNF. Brain-derived neurotrophic factor; IGF-1, insulin growth factor-1; FGF-1, fibroblast growth factor-1; MCI, mild cognitive impairment; RM, the maximum number of repetitions; VEGF, vascular endothelial growth factor; a, between-group differences; b, within-group differences;^*^: significant; NS, not specified.

### Effects of acute resistance exercise on cognitive function of healthy elderly people

With the increase of age, the cognitive flexibility required in the complex decision-making process is greatly reduced. However, resistance exercise can satisfy the functions of neurogenesis and neural circuits by supplying nutrition and energy (74), and provide multi-faceted health benefits for the body. In recent years, it has been confirmed that acute RE can improve cognitive executive function, including attention, working memory, problem-solving ability, cognitive flexibility, and verbal fluency. Chang et al. (76) conducted acute resistance exercise on 30 middle-aged and elderly people (7 actions, 2 groups, 70%1RM, 10 repetitions), and found that the performance of the exercise group under all Stroop tests was significantly improved compared with the control group. The study pointed out that acute resistance exercise can improve general cognition, but it is more beneficial to cognitive executive control ability. Hsieh et al. (77) observed from different age groups that acute moderate resistance exercise (70%1RM) is beneficial to the working memory of male adults (21–30 years old) and the elderly (65–72 years old), but it has a greater impact on the elderly when it comes to work tasks with higher memory requirements. Hsieh et al. (78) studied the difference in attention between male adults (21–30 years old) and the elderly (65–69 years old) in the same way of exercise and found that attention was improved after exercise, and pointed out that impact of acute resistance exercise on cognition would not be weakened with age.

However, acute resistance exercise may only have short-term benefits. Nicklas et al. (87) measured that the concentration of growth hormone GH in the elderly after acute resistance training increased by as much as 18 times compared with that before training, but after 10–15 min of training, these increased concentrations would return to the initial concentration value. Basso et al. (5) also found that acute isokinetic RE of lower limbs only temporarily increased the concentration of IGF-1 in peripheral blood, and the cognitive benefits could only be maintained for 2 h after exercise. Chang et al. (88) discussed the application time after the meta-analysis. If the cognitive task is applied within 5 min after exercise, the effect of exercise is negative (Cohen's *d* = −0.06), which may be due to the influence of exercise-induced tachycardia and hyperthermia on cognitive function. If the test time is 11–20 min or more after exercise, the effect of exercise is positive (Cohen's *d* = 0.26 or 0.17), which indicates that the benefit of acute resistance exercise on cognitive function comes into being about 10 min after the end of the exercise, and then this benefit gradually disappears (89). It is suggested that exercise intensity, amount of exercise, duration, evaluation of test time, and participants' health status are important factors that affect cognitive performance.

### Effects of chronic resistance exercise on cognitive function of healthy elderly people

Although the circulatory process (blood flow, hormones) seems to moderate the changes in cognitive ability caused by acute exercise, the long-term response may be explained by structural adaptation. It was found that after 52 weeks of resistance exercise, the concentration of IGF-1 in the peripheral serum of the elderly increased, and at the same time, the cognitive behavior (such as the accuracy and reaction time in the executive function test) and cognitive function (such as P3 amplitude) were improved (90). Coesee (85) found that during the Stroop test, the activation index of the prefrontal cortex decreased, the oxyhemoglobin and total hemoglobin of the left prefrontal cortex decreased (both of which were compared with the pre-test), and the cognitive task performance (i.e., reaction time) improved after 16 weeks of resistance exercise intervention for healthy elderly people. In another study of resistance exercise, it was found that after completing resistance exercise for up to 52 weeks, the performance of the Stroop test increased and the atrophy of cortical white matter decreased (compared with the elderly who performed balance and conditioning exercises). In addition, the study (84) also found that the decrease in brain activation was observed under relatively easy task conditions, while the increase in brain activation was found under more difficult task conditions.

Coelho et al. (80) proposed that chronic RE can trigger neurogenesis more. Experiments showed that the plasma BDNF of elderly women increased by 65.2% after three times a week of lower limb resistance exercise for 10 weeks. This is consistent with the results of other studies (82), which hypothesized that the increase of peripheral BDNF caused by resistance exercise would help to increase the resistance to age-induced brain damage and neurodegeneration. In addition, Chang et al. (91) pointed out that to significantly improve the cognitive function of the elderly (including information processing speed and attention) and maintain chronic benefits, it should be done at least twice a week for 2–12 months. Nagamatsu et al. (92) found that resistance exercise twice a week for 6 months (40% increased to 80%1RM) can enhance selective attention, conflict resolution, and associative memory of the elderly. Liu-Ambrose (86) also found that moderate and high-intensity resistance exercise (70–80%1RM, 6–8 repetitions) once or twice a week for 12 months can improve the above cognitive function. Then, Tsai et al. (83) randomly assigned 48 healthy old men to the resistance exercise group (75–80%1RM, 10 repetitions) or the control group, and carried out exercise training three times a week for 12 months. The results showed that the accuracy and reaction time of the exercise group were improved in the Oddball cognitive test, pointing out that moderate and high-intensity resistance exercise can help reduce the cognitive deterioration rate of the elderly.

## Discussion

This review is the first to summarize the available evidence for the acute effects of RE on cognitive function. Our findings demonstrate that both acute and chronic can increase GH and IGF-1 secretion and improve cognitive ability in the elderly. Because in the state of anaerobic exercise, it can stimulate vascular endothelial growth factor (VEGF) and pituitary gland to secrete GH, reduce the concentration of homocysteine and promote the liver to secrete IGF-1, increase c-Fos cells in the nerves, and trigger the activation and regeneration of neurons in the hippocampus, to improve the plasticity and memory function of synapses (55), which has positive effects on slowing down aging and improving memory function. Moreover, with aging, the oxidation-reduction state is out of balance due to immune system disorder. The reduction of antioxidant uptake results in the accumulation of active free radicals and a significant increase of pro-inflammatory factors in the body, such as interleukin-6(IL-6), IL-1β, and tumor necrosis factor (TNF-α). The anti-inflammatory factor IL-10 is significantly decreased compared with that in normal adults (93). The increase in inflammatory reaction and the production of reactive oxygen species may play an important role in the pathophysiological mechanism of MCI and AD. However, after the intervention of resistance exercise, peripheral or encephalitis cytokines can be down-regulated, delay telomere DNA damage and protein aggregation, inhibit mitochondrial dysfunction and the expression of pro-inflammatory factors in aging cells, reduce lipid peroxidation level, and regulate microglial activation (38), thereby preventing oxidation and inflammatory mechanisms from playing a role in aging and delaying or preventing the occurrence of MCI or AD.

For those who exercise regularly, their BDNF level is higher than those who exercise less in their lifestyle. The reason is that lactic acid released from the periphery (such as muscle tissue) after exercise can be used as a “fuel” for the cognitive process. With the help of monocarboxylic acid transporters, it passes through BBB (94, 95), triggering the activation of BDNF in the hippocampus and the release of BDNF in serum (96, 97), which is responsible for memory and learning, stimulating the function of the neural circuit and improving it. Importantly, BDNF produced after resistance exercise has direct or indirect interaction with IGF-1, that is, it can help the brain increase IGF-1, promote nerve growth and produce serum angiotensin and amino acid, and then stimulate more BDNF receptors to increase the connection between neurons. This neurobiochemical mechanism can act on such central nervous system regions as the forebrain, striatum, hippocampus, cerebral cortex, spetum neurons, cerebellum, and motor neurons (98). These brain structures are all related to cognitive processing.

We also found that the improvement effect of brain neurophysiology and cognitive function after a single acute resistance intervention was short-lived, and the accompanying time gradually disappeared. However, chronic resistance intervention can increase the concentration of IGF-1 in peripheral serum, increase the expression during the Stroop test, reduce the atrophy of white matter in the cerebral cortex, and improve the performance of cognitive tasks. Aleman et al. (99) recruited 25 elderly people with an average age of 69.1 years, who were in good health and had no mental or cognitive diseases, and tested them on eight cognitive tests (such as knowledge, language, visual-spatial perception, reading speed, structural organization, perceptual motor processing speed, cognitive processing speed, and long-term memory). The results showed that there was a high correlation between IGF-1 concentration and cognitive performance. The elderly with higher IGF-1 concentration had better cognitive performance. In addition, if the progressive moderate and high-intensity resistance exercise is used for more than 6 months, it can improve the selective attention, conflict resolution ability, and associative memory of healthy elderly people, while 12 months of moderate and high-intensity resistance exercise (70–80%1RM) can improve attention, accuracy, and reaction time. Therefore, acute resistance exercise may need to improve the cognitive flexibility and enhance the cognitive ability (such as working memory, attention reaction time, executive control ability, or information processing speed) of adults and the elderly in the long-term or in the case of moderate and high-intensity exercise, especially for the elderly. Compared with chronic resistance exercise, acute resistance seems to be inferior. However, the acute enhancement of cognitive function may have value in different situations. For example, the use of acute resistance exercise during athlete warm-up can play a role in sports injuries. Because in most cases of possible trauma, athletes need to quickly integrate and process large amounts of sensory information at the upper spine level, to develop and adjust exercise programs in time-tight situations. At the same time, help them make relevant decisions during the competition, and then improve their sports performance.

Finally, based on the above literature, the factors that affect the cognitive effect may be related to the intensity, amount, and duration of resistance exercise, and the difference between the evaluation time and the subjects. Importantly, these regulators do not influence the relationship between resistance exercise and cognitive function alone but interact with other regulatory factors to influence the cognitive benefits of resistance exercise. In a word, these results support the view that acute resistance usually has little effect on cognitive performance, and it may have great benefits when specific intervention measures and exercise parameters are needed. However, no matter what type of exercise (acute or chronic) is selected, it can cause changes in brain neuroplasticity, improve the cognitive function of healthy elderly people, and ensure that physical function and cognitive function can be maintained with age.

## Conclusion and further directions

### Conclusion

(1) Resistance training can have a beneficial effect on the brain function of the elderly population by changing blood flow, stimulating the metabolism of nerve conduction substances, neurotrophic substances, and endocrine hormones, promoting the neogenesis of cerebral vessels, and increasing the volume of gray matter in the brain, thereby improving the communication between muscle fibers stimulated by motor nerves and the brain, and preventing or delaying the cognitive deterioration of the brain during aging.
(2) Both acute resistance exercise and chronic resistance exercise can improve the cognitive function of the elderly, but acute resistance can only stimulate hormone secretion temporarily and improve the short-term recall effect, which has little effect on the cognitive performance of the elderly; Chronic resistance can significantly increase the secretion of nerve substances related to cognitive function in the brain of the elderly, such as hormones, neurons, growth factors, etc.
(3) It is suggested that gradual moderate and high-intensity resistance exercise (50–80%1RM, 1–3 times/week, 2–3 groups/time) be intervened for at least 6 months, which can significantly improve the working memory and cognitive processing speed the elderly, enhance cognitive behavior performance and achieve continuity effect.

### Further directions

(1) The decrease of brain activation caused by acute resistance or chronic resistance may be different from the neurobiological mechanism induced by acute aerobic or chronic aerobic. In the future, it is urgent to study the potential biological mechanism of cognitive function changes caused by different types of acute exercise and chronic exercise intervention to better understand the changes in brain function.
(2) The substantial correlation between hormone secretion and cognitive function induced by different intervention modes is also worthy of further study. Moreover, the research on different combination effects among variables such as training intensity, frequency, duration, and resistance is still scarce, which needs to be further clarified in the future.
(3) Moderate and high-intensity resistance exercise has certain risks to the elderly. Future research should pay more attention to individual differences, and the safety of resistance exercise prescription in different physical states should be given priority.

## Data availability statement

The original contributions presented in the study are included in the article/supplementary material, further inquiries can be directed to the corresponding author.

## Author contributions

AC was in charge of proposing topics and writing articles. JL was in charge of directing and revising articles, and the rest of the participants were in charge of collecting data, evaluating the quality of the literature, and drawing tables. All authors contributed to the article and approved the submitted version.

## Funding

This work was supported by the National Social Science Foundation of China (Grant No. 19ZDA352).

## Conflict of interest

The authors declare that the research was conducted in the absence of any commercial or financial relationships that could be construed as a potential conflict of interest.

## Publisher's note

All claims expressed in this article are solely those of the authors and do not necessarily represent those of their affiliated organizations, or those of the publisher, the editors and the reviewers. Any product that may be evaluated in this article, or claim that may be made by its manufacturer, is not guaranteed or endorsed by the publisher.
